# Influence of diet on leukocyte telomere length, markers of inflammation and oxidative stress in individuals with varied glucose tolerance: a Chinese population study

**DOI:** 10.1186/s12937-016-0157-x

**Published:** 2016-04-12

**Authors:** Meicen Zhou, Lixin Zhu, Xiangli Cui, Linbo Feng, Xuefeng Zhao, Shuli He, Fan Ping, Wei Li, Yuxiu Li

**Affiliations:** 1 Department of Endocrinology, Key Laboratory of Endocrinoly, Peking Union Medical College Hospital, Peking Union Medical College, Chinese Academy of Medical Sciences, Beijing, 100730 China; 2 Nankou Community Health Service Centers, Changping District, Beijing, 102200 China; 3 Nankou Railway Hospital, Changping District, Beijing, 102200 China; 4 Department of Nutrition, Peking Union Medical College Hospital, Peking Union Medical College, Chinese Academy of Medical Sciences, Beijing, 100730 China

**Keywords:** Leukocyte telomere length, Oxidative stress, Inflammation, Plasma glucose status, Carbohydrates/fat proportion, Diet ingredient

## Abstract

**Objectives:**

To explore influence of carbohydrates/fat proportions, dietary ingredients on telomere length shortening, oxidative stress and inflammation in a Chinese population with different glucose tolerance status.

**Methods:**

Five hundred and fifty-six Chinese subjects without diabetes history underwent a 75 g, 2 h Oral Glucose Tolerance Test (OGTT). Subjects with diabetes (*n* = 159), pre-diabetes (*n* = 197), and normal glucose tolerance (*n* = 200) were screened. Dietary intakes were evaluated using a semi-quantitative food frequency questionnaire (FFQ). Peripheral blood leukocyte telomere length (LTL) was assessed using a real-time PCR assay. Blood lipid profile, levels of the oxidative stress indicators superoxide dismutase (SOD), glutathione reductase (GR), 8-oxo-2′-deoxyguanosine (8-oxo-dG) and inflammation indicators tumor necrosis factor (TNF-ɑ), interleukine-6 (IL-6) were measured. Levels of HbA1c, plasma glucose, insulin, and C peptide were also determined. Measurements were taken at 0 min, 30 min, 60 min, and 120 min after 75 g OGTT. Insulin sensitivity was evaluated by HOMA-IR. Basal insulin secretion index (HOMA-β), early phase disposition index (DI_30_) and total phase disposition index (DI_120_) indicate insulin levels at different phases of insulin secretion.

**Results:**

In patients with newly diagnosed diabetes, LTL adjusted by age was longer in HbA1c < 7 % group (log (LTL):1.93 ± 0.25) than in HbA1c ≥ 7 % group (log (LTL):1.82 ± 0.29). LTL was not associated with daily energy intake, diet fat, carbohydrates and protein proportions. Multiple linear regression analysis indicated that legumes, nuts, fish and seaweeds were protective factors for LTL shortening, and sweetened carbonated beverage was a risk factor for LTL shortening ( legumes: β = 0.105, *p* = 0.018; nuts: β = 0.110, *p* = 0.011; fish: β = 0.118, *p* = 0.007; seaweeds: β = 0.116, *p* = 0.009; sweetened carbonated beverage: β = −0.120, *p* = 0.004 ). Daily energy intake was positively associated with TNF-ɑ, IL-6 (TNF-ɑ: *r* = 0.125, *p* = 0.006;IL-6: *r* = 0.092, *p* = 0.04). Fat, carbohydrate proportions were positively associated with TNF-ɑ (fat: *r* = 0.119, *p* = 0.008 ; carbohydrate: *r* = 0.094, *p* = 0.043). Seaweeds and dairy intake were negatively associated with 8-oxo-dG (seaweed: *r* = −0.496, *p* = 0.001;dairy: *r* = −0.246, *p* = 0.046 ), vegetables and fruits were positively associated with GR ( vegetables: *r* = 0.101, *p* = 0.034;fruits: *r* = 0.125, *p* = 0.045). Cereal, meat were positively associated with TNF-ɑ ( cereal: *r* = 0.091, *p* = 0.048 ; meat: *r* = 0.405, *p* = 0.009).

**Conclusion:**

Diabetes patients with better plasma glucose (HbA1c < 7 %) had longer LTL, LTL could reflect plasma glucose status in diabetes patients. LTL were probably not influenced by diet carbohydrates/fat proportions but was associated with diet ingredients. Diet ingredients significantly impacted on markers of inflammation and oxidative stress, which probably had an effect on LTL.

**Electronic supplementary material:**

The online version of this article (doi:10.1186/s12937-016-0157-x) contains supplementary material, which is available to authorized users.

## Introduction

Telomeres are essential and dynamic regulators of cellular life span and chromosome integrity in eukaryocyte, composed of guanine-rich sequence-TTAGGG [1, 2]. Telomeres in somatic human cells shorten by 30–200 bp in each cell division, and once shortened to a critical length, cells are triggered into replicative senescence, an irreversible cell cycle block in G0/G1 and are susceptible to undergo apoptosis when exposed to increased oxidative stress [3–5]. DNA telomere length is maintained mainly by telomerase, and regulated by pro-inflammation cytokines and oxidative stress [4, 6, 7]. The release of reactive oxygen species (ROS) and pro-inflammatory cytokines both induced by increased oxidative stress damage telomere DNA and eventually lead to telomere length shortening.

Short telomere length increases the risk of diabetes. Normoglycemic population with shorter telomere length had a high risk in developing diabetes [7–9]. Hyperglycemia, which increases oxidative stress, accelerates the telomere length shortening especially in islet β cell, causing β cell dysfunction and reduced insulin secretion [10]. Telomere is not only a predictor of diabetes, by regulating oxidative stress and β cell apoptosis, it also takes an important part in the mechanism of diabetes.

**Fig. 1:**
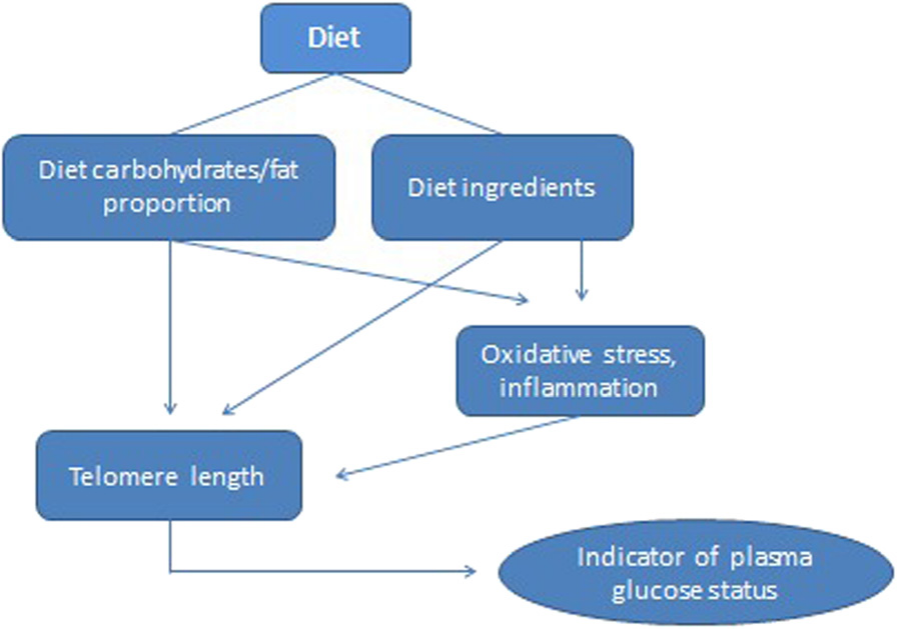
The hypothesis in the study

Energy intake and diet composition had an impact on DNA telomere length [11, 12], and oxidative stress involves in it. Animal experiments indicates that restricted calories intake in a short term increases the numbers of mitochondria and improves the function of respiratory chain, reducing the production of ROS, preventing telomere length shortening in the end [13, 14]. Diet composition also closely related with telomere length, diet can modulate telomerase activity in peripheral blood mononuclear cells [15, 16]. The pattern of Mediterranean diet mainly containing vegetables, legumes, fruits, grains, fish, single unsaturated fatty acids, dairy products, had a protective effect on telomere length [17, 18]. The medical nutrition therapy is a vital part in the treatment of diabetes, accumulating researchers found that dietary fat and carbohydrate proportions had an impact on plasma lipids, glucose control and inflammation in patients with type 2 diabetes [19, 20]. However, the associations between dietary carbohydrate/fat proportions and telomere length, oxidative stress have not been explored. There exists large differences of dietary composition in different regions in the world, Chinese dietary pattern is characteristic of low fat/high carbohydrate proportions, study was absent in the influence of dietary low fat/high carbohydrate proportions on the telomere length shortening and oxidative stress. The hypothesis of the study is that diet carbohydrate/fat proportion and diet ingredients may have a direct effect on telomere length or indirectly affect telomere length by regulating oxidative stress and inflammation status, telomere length could probably reflect plasma glucose status in population (Fig. 1).

The present study was to evaluate the telomere length, oxidative stress and inflammation status in a Chinese population with different glucose tolerance statuses, ranging from normoglycemia to pre-diabetes and diabetes and to explore influence of fat/carbohydrate proportions, dietary ingredients on telomere length shortening, oxidative stress, inflammation, and plasma glucose status.

## Methods

### Study population

All subjects were recruited from a type 2 diabetes project in a Beijing suburb in China between March 2014 and January 2015. Five hundred and ninety-nine subjects underwent a 75 g oral glucose tolerance test (OGTT). The 75 g OGTT was conducted after an overnight fast (>10 h). Blood samples were collected at 0 min, 30 min, 60 min and 120 min following the OGTT. The glucose tolerance status of each subject was classified based on the 1999 criteria of the WHO.

Subjects were excluded if they tested positive for Type 1 Diabetes Mellitus-related antibodies, were taking hypoglycemic or steroid drugs, or were taking drugs interfering with lipid metabolism. Subjects who had diabetes/impaired glucose tolerance history, cardiovascular diseases, cerebrovascular diseases, or nephropathy were also excluded. According to these criteria, subjects with NGT (*n* = 200), pre-diabetes (*n* = 197), and newly diagnosed diabetes (*n* = 159) were selected for this study (Additional file 1: Table S1). The study protocol was approved by the Ethics Committee of Peking Union Medical College Hospital. The subjects voluntarily signed informed consent forms.

### Dietary assessment

Information on dietary intake was collected using the semi-quantitative food frequency questionnaire (FFQ). This FFQ was based on the food frequency questionnaire used in the 2010 China National Nutrition and Health Survey (CNNHS). Information on frequency of intake and portion size was used to calculate the amount of each food item consumed on average, using China Food Composition Table (2009) as the database. The FFQ collects information on the average consumption frequency and serving size for 103 food items and beverage consumed in the previous year (the FFQ has been done in the latest three months in the year. During the three months, every week the trained investigator asked subjects to recall their food consumption in this week); there are nine categories of consumption frequency (‘almost never’, ‘once a month’, ‘2–3 times a month’, ‘1–2 times a week’, ‘3–4 times a week’, ‘5–6 times a week’, ‘once a day’, ‘twice a day’ or ‘3 times a day’) and three categories of serving size (‘larger than’, ‘equal to’ or ‘smaller than’ a standard serving size). During the interview using the FFQ, trained interviewers showed pictures of foods to the participants to help them estimate the serving size. To calculate the daily average consumption frequency of each food item, the frequency was multiplied by 1.5 for larger amounts, one for an equal amount and 0.5 for smaller amounts as compared with the standard serving size.

### Clinical measurement

A standardized medical history and accurate physical examination were undertaken in all of the subjects before a 75 g OGTT was administered. Measurements of waist circumference (WC) (midway between the iliac crest and the costal margin) and hip circumference (HC) (at the level of the trochanters) were performed twice by the same observer, and the mean value was recorded. Weight and height were measured without shoes in light clothing, and body mass index (BMI) was calculated by dividing the body weight in kilograms by the square of the height in meters. Blood pressure measurements from subjects at rest were obtained twice with a standard mercury sphygmomanometer, and the mean value was calculated.

### Biochemical measurements

Plasma glucose was measured by glucose oxidase assay. Cholesterol (TC), triglyceride (TG), high-density lipoprotein-cholesterol (HDL-C), and low-density lipoprotein-cholesterol (LDL-C) were determined using an automated analyzer. Serum insulin and C peptide were measured by chemiluminescent enzyme immunoassay. HbA1c analysis was performed by high-performance liquid chromatography (intra-assay CV <3 %, inter-assay CV <10 %). These biochemical indicators were measured among the whole study population.

### Assessment of insulin resistance (IR) and β cell function

Homeostatic model assessment of insulin resistance (HOMA-IR) was calculated to evaluate the IR [21].

The homeostasis model assessment of insulin secretion (HOMA-β) was calculated as basal insulin release [21]. Early-phase insulin release was calculated as the total insulin area under the curve divided by the total glucose area under the curve during the first 30 min of the OGTT (InsAUC_30_/GluAUC_30_). Insulin secretion relative to insulin sensitivity (ISI_M_) was expressed as the disposition index (DI), calculated as: early-phase DI_30_ = [InsAUC_30_/GluACU_30_] × ISI_M_, and total-phase DI_120_ = [InsAUC_120_/GluACU_120_] × ISI. The values calculated from the formulas above are highly correlated with first-phase insulin secretion in intravenous glucose tolerance test [22].

### Leukocyte DNA telomere length (LTL) measurement

All blood samples were preserved in −80^。^C refrigerator until they were tested. Genomic DNA in leukocytes was extracted from peripheral blood samples using the QIAamp DNA blood mid kit (Qiagen, Hilden, Germany). Purified DNA samples were diluted and quantified using a NanoDrop 1000 spectrophotometer (Thermo Fisher Scientific, Wilmington, DE, USA). Telomere length was determined as the relative ratio of telomere repeat copy number to the single copy number (T/S) using the novel monochrome multiplex quantitative PCR protocol described by Cawthon [23]. Telomere primer sequences were as follows:

- telg: 5′-ACACTAAGGTTTGGGTTTGGGTTTGGGTTTGGGTTAGTGT-3′,
- telc: 5′-TGTTAGGTATCCCTATCCCTATCCCTATCCCTATCCCTAACA-3′,

and albumin was employed as the single copy gene reference using primers modified with the addition of 5′-GC clamp to shift their melting temperature:

- albu: 5′-CGGCGGCGGGCGGCGCGGGCTGGGCGGAAATGCTGCACAGAATCCTTG-3′;
- albd: 5′-GCCCGGCCCGCCGCGCCCGTCCCGCCGGAAAAGCATGGTCGCCTGTT-3′.

The reagent components and final concentrations were 900 nM each primer (IDT), 1 × AmpliTaq Buffer II, 3 mM MgCl_2_, 0.2 mM per dNTP, 1 mM DTT, 1 M betaine, 0.75 × SYBR Green I and 0.625U AmpliTaq Gold DNA polymerase. Human genomic DNA samples, 5 ng to 70 ng, were used to generate two standard curves for each PCR plate (five concentrations with a high level of 150 ng and a low level of 1.85 ng per reaction). Thermal cycling: 1 cycle of 15 min at 95 °C; 2 cycles of 15 s at 94 °C, 15 s at 49 °C; and 32 cycles of 15 s at 94 °C, 10 s at 62 °C, 15 s at 74 °C with signal acquisition, 10 s at 84 °C, and 15 s at 88 °C with signal acquisition. Bio-Rad CFX Manger software automatically estimated the value for each sample T (telomere) and S(single copy gene) using standard curve. Standard curve efficiencies for both primer sets were above 90 %, and regression coefficients were at least 0.99 in all PCR runs. The within-plate and between-plate % coefficient of variation (%CV), which is based on the ratio of the standard deviation across replicates to the mean, were 18 and 7 %, respectively. For study samples, the with-plate %CV ranged from 8.2 to 14.3 %.

### Measurement of oxidative stress and inflammation indicators

Serum was collected from fasting blood samples. The levels of superoxide dismutase (SOD), glutathione reductase (GR), 8-oxo-2′-deoxyguanosine (8-oxo-dG), Tumor Necrosis Factor (TNF-ɑ), Interleukine-6 (IL-6) were determined as per the manufacturer’s instructions (Cloud-Clone Corp, Houston, USA). Absorbance kinetics were measured through an ELISA reader.

### Statistical analysis

All statistical analyses were performed using SPSS software, version 17.0 (Chicago, IL, USA). The data are presented as mean ± SD. Parameters not normally distributed were transformed. Categorical data were analyzed using the χ^2^ test. The significance of the mean difference was tested by ANOVA (followed by Bonferroni’s post hoc pairwise comparisons). Adjustment for covariates was performed with covariance analysis (ANCOVA). The LTL was adjusted by age and sex in the article. Pearson correlation was assessed between variables and risk factors. Stepwise multiple linear regression analysis was performed to exclude the influences of potential confounding variables between diet ingredients and LTL. All *P*-values were two-sided, and *P* < 0.05 was considered statistically significant.

## Results

### Clinical and demographic characteristics in groups with different DNA telomere length

There was no difference in LTL between women and men (women vs men: log (LTL): 1.98 ± 0.38 vs 1.95 ± 0.33, *p* = 0.327). Among the tertile groups of LTL, no difference was observed in age, sex, smoking, drinking, physical activity, BMI, WC, HC, blood pressure, HOMA-IR , HOMA-β, TG, HDL-C and LDL-C. HbA1c, FPG, PG 30′, PG 60′, PG120′ and TC were significantly higher in the lowest tertile group than those in the middle and upper tertile groups, and DI_30_ and DI_60_, which were both glucose-stimulated insulin secretion index, significantly decreased in the lowest tertile group compared with that in the middle and upper tertile groups (Table 1).

**Table 1:** Clinical and demographic characteristics in telomere length tertile groups

	Leukocyte telomere length			
	Short *N* = 184	Middle *N* = 186	Long *N* = 186	*P* value
Log(LTL)	1.61 ± 0.18	1.94 ± 0.08	2.23 ± 0.25	0.000**
Ages, years	52.92 ± 12.14	53.65 ± 11.63	53.08 ± 11.33	0.836
Sex (female), n(%)	114(61.96)	113(60.7)	116(62.37)	0.756
Current/Former smoking, n(%)	27(14.52)	29(15.93)	29(15.43)	0.625
Alcohol, n(%)	35(18.62)	38(20.88)	36(19.35)	0.658
Regular exercise, n(%)	69(37.10)	66(36.26)	71(37.77)	0.712
BMI, Kg/m^2^	25.60 ± 3.63	25.87 ± 3.81	26.08 ± 3.76	0.509
Waist circumference, cm	86.57 ± 9.89	87.29 ± 9.89	87.11 ± 9.51	0.786
Hip circumference, cm	92.29 ± 10.46	92.77 ± 11.25	92.48 ± 9.11	0.912
Systolic blood pressure, mmHg	126.04 ± 18.32	125.86 ± 19.07	129.56 ± 18.66	0.140
Diastolic blood pressure, mmHg	75.29 ± 9.65	76.61 ± 9.60	76.74 ± 10.45	0.340
Log(8-oxo-dG, pg/ml)	5.06 ± 1.17	5.02 ± 1.04	4.98 ± 1.02	0.767
SOD, U/ml	56.63 ± 14.01	58.41 ± 18.26	63.07 ± 15.34	0.001**
GR, U/L	7.13 ± 3.18	6.98 ± 3.118	6.82 ± 3.12	0.687
Log(TNF-ɑ, fmol/ml)	4.64 ± 0.52	4.29 ± 0.66	4.19 ± 0.74	0.000**
Log(IL-6, pg/ml)	1.69 ± 0.95	1.68 ± 0.91	1.51 ± 0.90	0.173
HbA1c%	5.94 ± 1.21	5.92 ± 1.11	5.56 ± 0.46	0.001**
FPG, mmol/L	6.60 ± 2.13	6.58 ± 1.83	5.88 ± 0.62	0.000**
PG 30′ , mmol/L	11.32 ± 3.39	10.92 ± 3.47	9.88 ± 2.47	0.000**
PG 60′ , mmol/L	10.98 ± 4.80	10.85 ± 4.81	9.43 ± 3.51	0.003**
PG 120′ , mmol/L	9.13 ± 5.15	8.82 ± 4.88	7.53 ± 2.95	0.004**
Ln (Ins 0′ , mU/L)	2.27 ± 0.61	2.28 ± 0.58	2.30 ± 5.41	0.902
Ln (Ins 30′ , mU/L)	3.99 ± 0.76	4.01 ± 0.73	4.27 ± 0.79	0.002**
Ln (Ins 60′ , mU/L)	4.06 ± 0.71	4.11 ± 0.71	4.22 ± 0.78	0.157
Ln (Ins 120′ , mU/L)	3.66 ± 0.82	3.70 ± 0.79	3.80 ± 0.85	0.347
Ln (CP 0′ , ng/mL)	0.25 ± 0.46	0.30 ± 0.42	0.34 ± 0.75	0.338
Ln (CP 30′ , ng/mL)	1.40 ± 0.55	1.43 ± 0.55	1.64 ± 0.91	0.003**
Ln (CP 60′ , ng/mL)	1.59 ± 0.54	1.79 ± 0.50	1.86 ± 0.61	0.004**
Ln (CP 120′ , ng/mL)	1.57 ± 0.49	1.59 ± 0.49	1.70 ± 0.59	0.077
Ln (HOMA-IR)	1.37 ± 0.51	1.36 ± 0.49	1.30 ± 0.38	0.373
Sqrt (HOMA-β)	8.91 ± 2.82	8.93 ± 2.92	9.50 ± 2.52	0.104
Sqrt (DI 30)	18.34 ± 6.84	19.96 ± 2.96	21.66 ± 6.13	0.001**
Sqrt (DI 120)	22.41 ± 6.93	22.43 ± 6.94	24.98 ± 5.27	0.000**
Log (TG, mmol/L)	0.55 ± 1.03	0.48 ± 0.85	0.43 ± 1.29	0.409
TC, mmol/L	5.63 ± 1.09	5.41 ± 1.11	5.32 ± 1.12	0.038*
Log(HDL-C, mmol/L)	0.34 ± 0.35	0.35 ± 0.34	0.41 ± 0.59	0.299
LDL-C, mmol/L	2.76 ± 0.75	2.78 ± 0.76	2.90 ± 0.73	0.195

*p < 0.05, **p < 0.01

Subjects were divided into three groups according to their plasma glucose levels: NGT, pre-diabetes, or newly diagnosed diabetes, LTL was longest in the NGT, it was shortest in diabetes, and LTL in pre-diabetes was in the middle (log (LTL): NGT vs pre-diabetes vs newly diagnosed diabetes: 2.01 ± 0.03 vs 1.97 ± 0.03 vs 1.89 ± 0.03, *p* = 0.005).

The newly diagnosed diabetes was divided into two groups: HbA1c <7 % group and HbA1c ≥ 7 % group. LTL was much longer and WC was smaller in HbA1c <7 % group than in HbA1c ≥ 7 % group, there was no significant difference in age, BMI, HC, blood pressure, smoking, drinking and physical activity between the two groups (Table 2).

**Table 2:** Characteristics between different glucose control groups in newly diagnosed diabetes

	HbA1c <7 % (*n* = 84)	HbA1c ≥ 7 % (*n* = 75)	*p* value
HbA1c%	6.07 ± 0.52	8.40 ± 1.38	0.000**
Ages, years	58.0 ± 9.8	57.6 ± 11.1	0.767
Sex (female), n(%)	52 (61.90)	46 (61.33)	0.879
Current/Former smoking, n(%)	5 (5.95)	3 (4.00)	0.638
Alcohol, n(%)	11(13.09)	10(13.33)	0.815
Regular exercise, n(%)	56(66.67)	48(64.00)	0.807
BMI, Kg/m^2^	25.92 ± 3.68	26.29 ± 3.94	0.500
Waist circumference, cm	86.93 ± 7.59	89.96 ± 10.89	0.023*
Hip circumference, cm	93.54 ± 10.57	96.63 ± 11.75	0.052
Systolic blood pressure, mmHg	131.47 ± 20.16	131.65 ± 18.36	0.948
Diastolic blood pressure, mmHg	76.08 ± 10.15	76.77 ± 10.49	0.635
Log(TL)	1.93 ± 0.25	1.82 ± 0.29	0.022*
Log(8-oxo-dG, pg/ml)	5.21 ± 1.02	5.40 ± 1.06	0.207
SOD, U/ml	60.03 ± 14.46	57.52 ± 10.01	0.182
GR, U/L	6.40 ± 3.21	6.27 ± 2.65	0.774
Log(TNF-ɑ, fmol/ml)	3.46 ± 0.62	4.33 ± 0.64	0.031*
Log(IL-6, pg/ml)	1.63 ± 0.83	1.86 ± 0.91	0.568
Daily energy intake, kcal	1441.86 ± 631.64	1554.24 ± 556.51	0.340
Daily protein intake, g	45.68 ± 19.84	50.59 ± 24.09	0.138
Daily fat intake, g	45.83 ± 13.06	46.42 ± 18.23	0.908
Daily carbohydrate intake, g	252.55 ± 121.06	257.36 ± 123.41	0.794
Protein % of energy intake	10.74 ± 5.44	9.72 ± 7.25	0.256
Fat % of energy intake,%	23.14 ± 8.99	22.26 ± 9.80	0.515
Carbohydrate % of energy intake	66.12 ± 15.74	68.02 ± 26.84	0.062
Daily cereal and cereal production intake, g	281.24 ± 37.10	288.56 ± 38.68	0.718
Daily tuber crop intake, g	19.72 ± 12.63	23.14 ± 10.18	0.470
Daily legumes product intake, g	64.00 ± 10.92	86.67 ± 11.88	0.187
Daily meat intake, g	70.50 ± 16.69	125.71 ± 12.37	0.212
Daily dairy products intake, g	258.71 ± 31.83	246.88 ± 35.93	0.950
Seeds or nuts, g	45.83 ± 21.31	50.79 ± 14.25	0.784
Vegetables, g	106.50 ± 87.03	188.67 ± 97.91	0.518
Fruits, g/day	123.17 ± 36.52	126.25 ± 31.97	0.636
Fish and other seafood, g/day	43.75 ± 16.05	58.33 ± 34.16	0.381
Seaweed, g/day	63.08 ± 18.51	58.50 ± 15.69	0.686
Sweetened carbonated beverage, ml/day	150.00 ± 80.00	225.00 ± 35.35	0.003**

*p < 0.05, **p < 0.01

In the whole population, LTL was negatively associated with HbA1c, PG 30′, PG 60′ and PG 120′, and was positively with glucose-stimulated insulin release-DI_30_ and DI_60_. LTL was not related with HOMA-IR, HOMA-β, TC, TG, HDL-C and LDL-C (Table 3).

**Table 3:** Correlation of age-adjusted telomere length with metabolic risk factors

Parameters	Telomere length	
	*r* value	*p* value
Body mass index (BMI)	−0.004	0.930
Waist circumference (WC)	−0.019	0.672
Hip circumference (HC)	−0.007	0.878
Systolic blood pressure	0.025	0.585
Diastolic blood pressure	−0.018	0.693
HbA1c	−0.139	0.002**
Fasting plasma glucose (FPG)	−0.080	0.077
Postprandial plasma glucose (PG) 30′	−0.186	0.000**
PG 60′	−0.162	0.000**
PG 120′	−0.155	0.001**
Ln (Insulin 0′)	−0.022	0.629
Ln (Insulin 30′)	0.065	0.154
Ln (Insulin 60′)	0.012	0.792
Ln (Insulin 120′)	−0.042	0.357
Ln (C peptide 0′)	−0.025	0.586
Ln (C peptide 30′)	0.040	0.384
Ln (C peptide 60′)	−0.015	0.743
Ln (C peptide 120′)	−0.059	0.192
Ln HOMA-IR	−0.051	0.265
Sqrt HOMA-β	0.003	0.954
Sqrt DI 30	0.139	0.002**
Sqrt DI 120	0.108	0.018*
TC	0.002	0.968
Log (TG)	0.021	0.646
Log (HDL-C)	−0.050	0.273
LDL-C	0.032	0.475
Daily energy intake, kcal	−0.003	0.946
Daily protein intake, g	−0.006	0.910
Daily fat intake, g	−0.044	0.391
Daily carbohydrate intake, g	−0.019	0.704
Protein % of energy intake	−0.023	0.622
Fat % of energy intake,%	0.047	0.326
Carbohydrate % of energy intake	−0.022	0.628
Daily cereal and cereal production intake, g	−0.037	0.465
Daily tuber crop intake, g	−0.025	0.627
Daily legumes product intake, g	0.297	0.000**
Daily meat intake, g	−0.007	0.922
Daily dairy products intake, g	0.228	0.152
Seeds or nuts,g	0.270	0.000**
Vegetables, g	0.058	0.311
Fruits, g/day	−0.088	0.255
Fish and other seafood, g/day	0.343	0.000**
Seaweed, g/day	0.251	0.006**
Sweetened carbonated beverage, ml/day	−0.268	0.000**

*p < 0.05, **p < 0.01

### Association between diet carbohydrate/fat proportions and LTL, oxidative stress, inflammation indicators

**Fig. 2:**
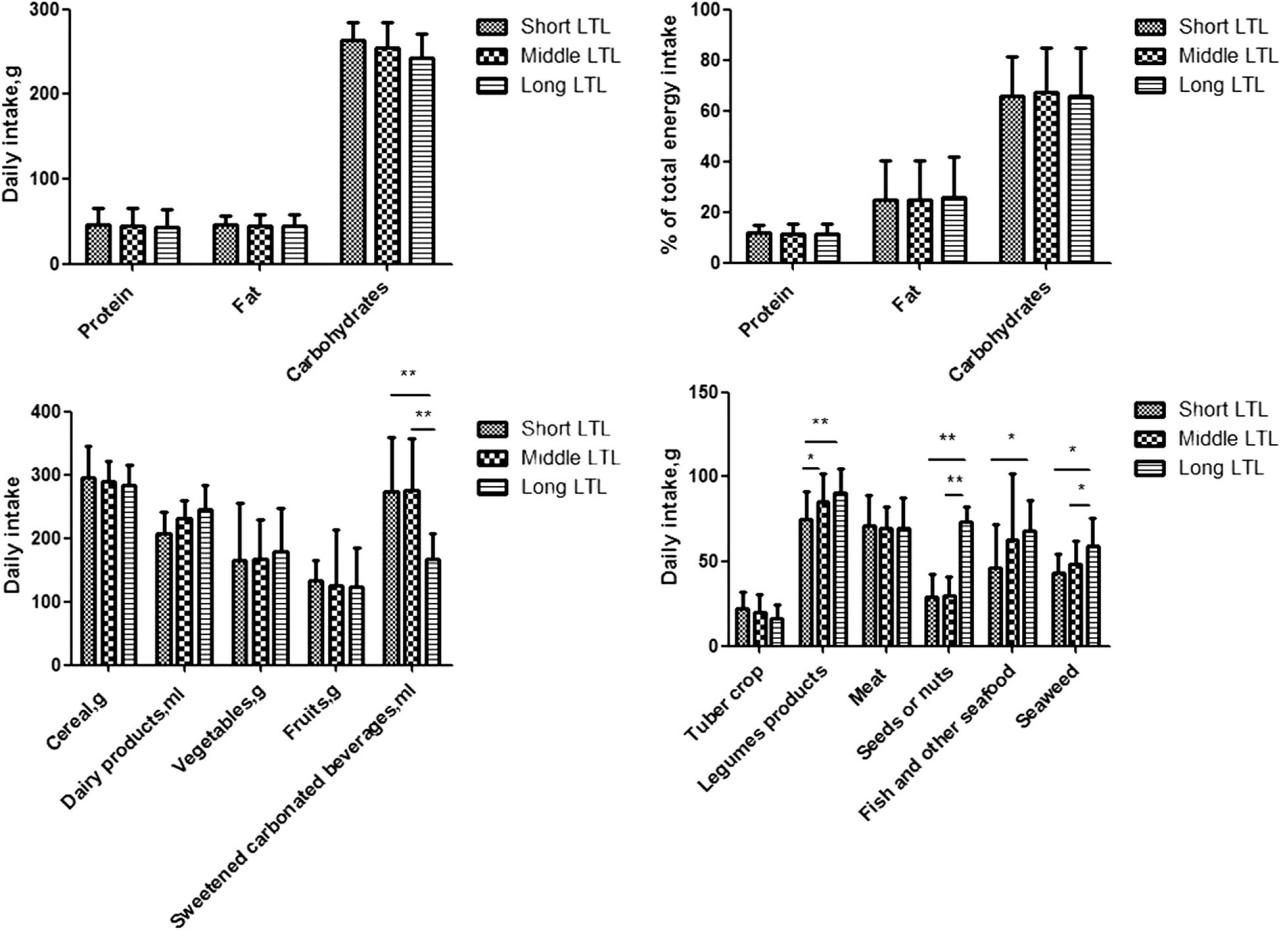
*p < 0.05, **p < 0.01. Comparison among the tertile groups of telomere length: **a** shows the daily intake of protein, fat and carbohydrates. **b** shows the percentage of protein, fat, carbohydrate account for the daily total energy intake. **c** shows the daily intake of cereal, dairy products, vegetables, fruits and sweetened carbonated beverage. **d** shows the daily intake of tuber crop, legumes, meat, seeds or nuts, fish seafood and seaweeds

Among the tertile groups of LTL, no difference was observed in daily energy intake (shortest vs middle vs longest: 1604.10 ± 633.15 vs 1598.33 ± 754.29 vs 1499.05 ± 599.98(kcal), *p* = 0.333), and there was no significantly difference in the fat, carbohydrate, protein intakes and their intake proportions (Fig. 2a, b).

Correlation analysis showed that daily energy intake and fat, carbohydrate, protein proportions was not associated with LTL (Table 3). Daily energy intake was positively associated with TNF-ɑ, IL-6 (TNF-ɑ: *r* = 0.125, *p* = 0.006;IL-6: *r* = 0.092, *p* = 0.04), and it was not related with 8-oxo-dG, SOD and GR (8-oxo-dG:*r* = 0.040, *p* = 0.388; SOD: *r* = −0.007, *p* = 0.873;GR:*r* = −0.023, *p* = 0.611). Fat, carbohydrate proportions were positively associated with TNF-ɑ (fat: *r* = 0.119, *p* = 0.008 ; carbohydrate: *r* = 0.094, *p* = 0.043), and were not related with 8-oxo-dG, SOD, GR and IL-6 (Additional file 1: Table S2).

### Association between diet ingredients and LTL, oxidative stress, inflammation indicators

LTL was adjusted by daily energy intake. Among the tertile groups of LTL, there was no significant difference in the intake of cereal, tuber, meat, vegetable, fruits and dairy production. Legumes, seeds or nuts, fish and seaweeds intakes were higher, and sweetened carbonated beverage (including sugar-sweetened beverage such as sucrose or fructose maize syrup and artificially sweetened beverage such as aspartame) intake was less in the upper tetile group than in the middle and lowest tertile groups (Fig. 2c, d).

Correlation analysis showed that LTL was not associated with cereal, tuber, meat, vegetables, fruits and dairy production, but it was positively related with legumes, nuts, fish, seaweeds and was negatively related with sweetened carbonated beverage intake (Table 3). Seaweeds and dairy intake were negatively associated 8-oxo-dG (seaweed: *r* = −0.496, *p* = 0.001;dairy: *r* = −0.246, *p* = 0.046), vegetables and fruits were positively associated with GR (vegetables: *r* = 0.101, *p* = 0.034;fruits: *r* = 0.125, *p* = 0.045), cereal and meat were positively associated with TNF-ɑ (cereal: *r* = 0.091, *p* = 0.048 ; meat: *r* = 0.405, *p* = 0.009). Diet ingredients were not significantly related with IL-6 (Additional file 1: Table S2).

Multiple linear regression analysis indicated that legumes, nuts, fish and seaweeds were protective factors for LTL (legumes: β = 0.105, *p* = 0.018; nuts: β = 0.110, *p* = 0.011; fish: β = 0.118, *p* = 0.007 ; seaweeds: β = 0.116, *p* = 0.009), and sweetened carbonated beverage was a risk factor for LTL adjusted by HbA1c, SOD, GR, IL-6, TNF-ɑ, 8-oxo-dG (Sweetened carbonated beverage: β = −0.120, *p* = 0.004 ).

### Carbohydrate/fat proportions, diet ingredients related with plasma glucose profiles in the whole population

Daily diet carbohydrate/fat proportions were positively associated with HbA1c, FPG ( HbA1c: carbohydrate: *r* = 0.168, *p* = 0.000;fat: *r* = 0.186, *p* = 0.000; FPG: carbohydrate: *r* = 0.206, *p* = 0.000;fat: *r* = 0.138, *p* = 0.001 ). Diet protein proportion was not related with HbA1c, FPG. After adjusted by daily energy intake, sweetened carbonated beverage was positively associated with HbA1c (*r* = 0.100, *p* = 0.022), FPG (*r* = 0.113, *p* = 0.010). There was no significant relation between cereal, tuber, legumes, meat, nuts, vegetables, fruits, fish, seaweed, dairy and HbA1c, FPG (Additional file 1: Table S3).

### Diet ingredients, LTL, inflammation indicators related with plasma glucose profiles in newly diagnosed diabetes

In patients with newly diagnosed diabetes, daily sweetened carbonated beverage intake was less in HbA1c <7 % group than in HbA1c ≥ 7 % group. LTL was much longer in HbA1c <7 % group than in HbA1c ≥ 7 % group. There was no significant difference in daily energy intake, carbohydrate/fat proportions, cereal, tuber, legumes, meat, nuts, vegetables, fruits, fish, seaweed and dairy. TNF-ɑ was lower in HbA1c <7 % group than in HbA1c ≥ 7 % group, there was no difference in SOD, GR, 8-oxo-dG and IL-6 between the two groups (Table 2).

## Discussion

The nutrition therapy takes a vital part both in the prevention and the treatment of diabetes by improving glucose control and cardiovascular risk factors [24, 25]. Restricted calories intake and low carbohydrate/low fat diets ameliorate plasma lipids, glucose and inflammation status, however, the distribution of diet carbohydrate/fat proportions in the nutrition therapy remains to be debated [20, 26, 27]. Compared with western population, diet carbohydrate proportion was relatively higher and fat proportion was lower in Chinese population, in the present study carbohydrate intake accounted for 67.60 ± 18.92 % of daily energy intake, much higher than recommended carbohydrate proportion (45–60 % of energy intake) according to American Diabetes Association (ADA) [28]. The fat intake in the study was 22.09 ± 16.43 % of energy intake, less than recommended fat proportion (25–35 % of energy intake) [28]. Therefore, the population in the study was in a high carbohydrate/low fat proportion dietary pattern. The present study found that in Chinese population with high carbohydrate/low fat proportion intake, diet carbohydrate/fat proportions were not significantly associated with LTL and oxidative stress indicators, suggesting that LTL were probably not influenced by diet carbohydrate/fat proportions.

This study based on Chinese population with different glucose status, and adjusted by daily energy intake because energy intake had an impact on LTL [11], which was a confounder. Though in this study LTL was not related with carbohydrate/fat proportions, the results showed that LTL was associated with diet ingredients, much more intake of legumes, nuts, fish were related with longer LTL, but much more intake of sweetened carbonated beverage was related with shorter LTL. Although the potential biological mechanisms underlying the associations between food items and LTL need to be explored, one possible explanation is that the nutrition composition has an influence on LTL. Previous study showed that the impact of dietary patterns on LTL resulted from the food item containing antioxidant and anti-inflammatory ingredients. Legumes contains a variety of planet chemicals such as isoflavones as an antioxidant, as well as folic acid, which play an important role in DNA methylation and integrity [29]. Nuts and fish containing polyunsaturated fatty acids, are the main food sources of omega-3 fatty acids and vitamin E, and have a protective function on LTL [30]. Fish also contains vitamin D, the anti-inflammatory and antiproliferative properties of vitamins D limit the turnover of cells, thus potentially reducing their telomere length attrition [16]. Seaweeds contain antioxidative enzymes to inhibit DNA damage [31]. In recent years, tradition prudent Chinese diet pattern has been affected by western diet pattern, more intake of sugar sweet drinks increased the sugar intake in Chinese diet, excessive intake of sweet drinks can increase energy intake, insulin resistance, oxidative stress and inflammation, accelerating LTL shortening, and regular consumption of sugar-sweetened beverage might influence metabolic disease development through accelerated cell aging [32, 33]. Previous studies mostly focused on diabetes population, which found that diet carbohydrate/fat proportions were associated with plasma glucose in diabetes patients [19], however, given that the diet pattern in diabetes patients to a certain extent influenced by diabetes education, it exists a certain deviation. Therefore, this study focused on newly diagnosed pre-diabetes and diabetes, and also contained normal glucose tolerance, in the population with different glucose tolerance, diet carbohydrate/fat proportions were positively associated with HbA1c, FPG, whether in impaired glucose tolerance or in normal glucose tolerance, increased diet carbohydrate/fat proportions could increase the occurrence of hyperglycemia.

Telomere is probably a mediator between life style and aging related diseases. In type 2 diabetes, telomere length shortening causes aging in islet β cell in advance, resulting in reduction of β cell mass and insulin release [7]. The present study suggested that shorter LTL was associated with higher HbA1c, FPG, postprandial glucose and lower glucose-stimulated insulin release. LTL shorten existed in pre-diabetes, and LTL was shortest in diabetes. According to previous longitudinal studies, shorter telomere length could predict the risk of diabetes, telomere length was not only an indicator of diabetes, also involves in the mechanism of diabetes [7, 9]. Moreover, LTL was closely associated with plasma glucose status in diabetes patients, hyperglycemia increased oxidative stress, and accelerated LTL shortening [34]. The results in this study found that in newly diagnosed diabetes patients with HbA1c < 7 % had longer LTL than the patients with HbA1c ≥ 7 %, it indicated that LTL could also reflect the plasma glucose status in diabetes patients.

The results in this study indicated that daily energy intake was positively associated with TNF-ɑ and IL-6, diet carbohydrate/fat proportions, cereals and meat intake were positively associated TNF-ɑ. Previous studies indicated that TNF-ɑ, IL-6 inflammatory signaling could affect cellular pathway (Iκ kinase β and Jun N-terminal kinase), reduce insulin sensitivity and regulate plasma glucose [35, 36], it was speculated that diet carbohydrate/fat proportions could regulate plasma glucose by inflammatory signaling pathway. It was suggested that diet probably regulated plasma glucose probably by inflammatory signaling pathway.

The study had some limitations. This study, which was a cross-sectional study to explore the association between LTL and diet proportions, cannot explain whether diet factors could affect the shorten rate of LTL, longitudinal study was more convincing than cross-sectional study. The sample size was relatively small, though the population in the study had a wide plasma spectrum. Another limitation was that although diet composition investigation questionnaire was based on the Chinese food ingredients scale, the investigators were trained, the validity and reliability can be guaranteed, however, some measurement errors in cannot be ruled out. The subjects including in the study were with relatively high BMI and high systolic blood press, this probably was a selection bias in the study.

## Conclusion

The present study in Chinese population with different plasma glucose status found that LTL were probably not influenced by diet carbohydrates/fat proportions but was associated with diet ingredients. Diet ingredients significantly impacted on markers of inflammation and oxidative stress, which probably had an effect on LTL. Diabetes patients with good plasma glucose had longer LTL, LTL could reflect plasma glucose status in diabetes patients.

## Additional file

Additional file 1:
**Table S1.** Characteristics of different glucose tolerance statuses. **Table S2.** Correlation of carbohydrate/fat/protein proportion with oxidative stress and inflammatory indicators. **Table S3.** Correlation of diet ingredients with HbA1c, FPG. (DOCX 20 kb)
