# Supplementary material for: Influence of diet on leukocyte telomere length, markers of inflammation and oxidative stress in individuals with varied glucose tolerance: a Chinese population study
Source: Nutr J. 2016 Apr 12;15:39. doi: 10.1186/s12937-016-0157-x (PMC4830058; doi:10.1186/s12937-016-0157-x)
Supplement: Additional file 1: — Table S1. Characteristics of different glucose tolerance statuses. Table S2. Correlation of carbohydrate/fat/protein proportion with oxidative stress and inflammatory indicators. Table S3. Correlation of diet ingredients with HbA1c, FPG. (DOCX 20 kb) [file 12937_2016_157_MOESM1_ESM.docx]

**Additional file 1**

**Table S1. Characteristics of different glucose tolerance statuses**

| **Parameters** | **NGT**  **n=200** | **Pre-diabetes n=197** | **Diabetes n=159** | **P value** |
| --- | --- | --- | --- | --- |
| **Age , years** | 48.73±12.06 | 55.34±10.24 | 57.80±10.42 | 0.000** |
| **Sex (male: female)** | 61:141 | 77:127 | 72:128 | 0.000** |
| **Body mass index (BMI), kg/m^2^** | 25.09±3.44 | 26.66±3.78 | 26.10±3.80 | 0.000** |
| **Waist circumference ,cm** | 84.69±9.70 | 88.73±9.29 | 88.36±9.39 | 0.000** |
| **Hip circumference ,cm** | 90.23±9.88 | 93.77±9.09 | 95.00±11.22 | 0.000** |
| **Systolic blood pressure (SBP) , mm Hg** | 123.38±18.73 | 129.06±16.16 | 131.55±19.29 | 0.000** |
| **Diastolic blood pressure (DBP), mm Hg** | 75.66±9.82 | 76.73±9.96 | 76.40±10.29 | 0.553 |
| **HbA1c (%)** | 5.27±0.29 | 5.71±0.33 | 7.19±1.55 | 0.000** |
| **Fasting plasma glucose (FPG) ,mmol/L** | 5.45±0.36 | 6.10±0.48 | 8.89±3.30 | 0.000** |
| **Postprandial plasma glucose(PG) 30’ ,mmol/L** | 8.97±1.91 | 10.71±2.02 | 14.16±4.44 | 0.000** |
| **PG 60’ ,mmol/L** | 7.60±1.98 | 10.09±2.78 | 16.11±5.13 | 0.000** |
| **PG 120’ ,mmol/L** | 5.82±1.21 | 7.61±1.75 | 14.68±5.68 | 0.000** |

*P <0.05, **P <0.01.

**Table S2. Correlation of carbohydrate/fat/protein proportion with oxidative stress and inflammatory indicators**

|  | ***r* value** | ***p* value** |
| --- | --- | --- |
| **Carbohydrate % of energy** |  |  |
| **8-oxo-dG** | 0.022 | 0.621 |
| **SOD** | -0.018 | 0.682 |
| **GR** | -0.033 | 0.463 |
| **TNF-ɑ** | 0.094 | 0.043* |
| **IL-6** | 0.001 | 0.976 |
| **Fat % of energy** |  |  |
| **8-oxo-dG** | 0.013 | 0.775 |
| **SOD** | 0.004 | 0.930 |
| **GR** | 0.086 | 0.051 |
| **TNF-ɑ** | 0.119 | 0.008** |
| **IL-6** | 0.014 | 0.753 |
| **Protein % of energy** |  |  |
| **8-oxo-dG** | 0.039 | 0.387 |
| **SOD** | -0.012 | 0.792 |
| **GR** | -0.066 | 0.133 |
| **TNF-ɑ** | 0.012 | 0.795 |
| **IL-6** | 0.008 | 0.853 |
|  |  |  |
|  |  |  |

**p*<0.05, **p<0.01.

**Table S3. Correlation of diet ingredients with HbA1c, FPG**

|  | ***r* value** | ***p* value** |
| --- | --- | --- |
| **HbA1c** |  |  |
| **Daily cereal and cereal production intake, g** | 0.058 | 0.189 |
| **Daily tuber crop intake, g** | -0.023 | 0.600 |
| **Daily legumes product intake, g** | -0.025 | 0.759 |
| **Daily meat intake, g** | 0.042 | 0.505 |
| **Daily dairy products intake, g** | 0.089 | 0.460 |
| **Seeds or nuts, g** | 0.015 | 0.899 |
| **Vegetables, g** | 0.014 | 0.756 |
| **Fruits, g/day** | 0.026 | 0.556 |
| **Fish and other seafood, g/day** | -0.064 | 0.318 |
| **Seaweed, g/day** | -0.032 | 0.654 |
| **FPG** |  |  |
| **Daily cereal and cereal production intake, g** | 0.026 | 0.556 |
| **Daily tuber crop intake, g** | 0.028 | 0.525 |
| **Daily legumes product intake, g** | 0.015 | 0.846 |
| **Daily meat intake, g** | 0.042 | 0.504 |
| **Daily dairy products intake, g** | 0.044 | 0.713 |
| **Seeds or nuts, g** | 0.062 | 0.220 |
| **Vegetables, g** | 0.048 | 0.672 |
| **Fruits, g/day** | 0.036 | 0.555 |
| **Fish and other seafood, g/day** | -0.017 | 0.876 |
| **Seaweed, g/day** | 0.012 | 0.866 |
